# Meta-analytic evidence that mindfulness training alters resting state default mode network connectivity

**DOI:** 10.1038/s41598-022-15195-6

**Published:** 2022-07-18

**Authors:** Hadley Rahrig, David R. Vago, Matthew A. Passarelli, Allison Auten, Nicholas A. Lynn, Kirk Warren Brown

**Affiliations:** 1 Department of Psychology, Virginia Commonwealth University, 806 W. Franklin Street, Richmond, VA 23284 USA; 2 Department of Psychology, Vanderbilt Brain Institute, Vanderbilt University, Nashville, USA TN

**Keywords:** Neuroscience, Cognitive neuroscience, Neural circuits

## Abstract

This meta-analysis sought to expand upon neurobiological models of mindfulness through investigation of inherent brain network connectivity outcomes, indexed via resting state functional connectivity (rsFC). We conducted a systematic review and meta-analysis of rsFC as an outcome of mindfulness training (MT) relative to control, with the hypothesis that MT would increase cross-network connectivity between nodes of the Default Mode Network (DMN), Salience Network (SN), and Frontoparietal Control Network (FPCN) as a mechanism of internally-oriented attentional control. Texts were identified from the databases: MEDLINE/PubMed, ERIC, PSYCINFO, ProQuest, Scopus, and Web of Sciences; and were screened for inclusion based on experimental/quasi-experimental trial design and use of mindfulness-based training interventions. RsFC effects were extracted from twelve studies (mindfulness *n* = 226; control *n* = 204). Voxel-based meta-analysis revealed significantly greater rsFC (MT > control) between the left middle cingulate (*Hedge’s g* = .234, *p* = 0.0288, *I*^2^ = 15.87), located within the SN, and the posterior cingulate cortex, a focal hub of the DMN. Egger’s test for publication bias was nonsignificant, bias = 2.17, *p* = 0.162. In support of our hypothesis, results suggest that MT targets internetwork (SN-DMN) connectivity implicated in the flexible control of internally-oriented attention.

## Introduction

Human waking life contains many moments in which the mind is not engaged by external goals or tasks, but is instead absorbed in a state of stimulus-independent thought (SIT)^1,2^, commonly referred to as mind wandering (MW). The definition of MW is multidimensional, and has previously been described as thought that is task-unrelated^3^, spontaneously unfolding^4^, or perceptually decoupled^5^ (i.e., “internally-oriented” or self-reflective in nature^6,7^). The content and dynamics of MW have been shown to vary considerably between individuals^8,9^, with important implications for mental health. While MW appears to serve multiple adaptive functions, including self-regulation^10^, memory consolidation^11^ (but see Refs.^12,13^), and problem solving^14,15^, the resting mind can paradoxically become restless in nature when thoughts are negatively-oriented, repetitive, or intrusive^5,16,17^. Such perseverative cognitions reinforce maladaptive coping strategies endemic to affective disorders (e.g., anxiety and depressive conditions)^18–21^.

The disruption of repetitive negative thinking is notoriously challenging^21,22^; however, research suggests that cognitive training in mindful awareness may alter the resting mind so as to function more adaptively^23,24^. The effects of mindfulness training for the treatment of affective disorders and concomitant rumination are well-documented^25–28^; however, the mechanistic involvement of resting state activity is less well-understood. Prevailing theory posits that mindfulness training may alter such activity via attentional mechanisms, ostensibly reflected in the reorganization of neural circuitry^24,29,30^. Accordingly, recent research has begun to investigate resting state neural circuitry as an outcome of mindfulness training (e.g., Refs.^31,32^) and as a characteristic of dispositional mindfulness (e.g., Refs.^33–36^). Such studies support the involvement of candidate interacting brain networks—including the default mode network (DMN), salience network (SN), and the frontoparietal control network (FPCN)—through which mindfulness may facilitate the flexible allocation of attentional resources between introspective and perceptual processes^37^. However, there remains little consensus about how mindfulness alters functional connectivity within and between these networks. The current meta-analysis sought to update brain-based models of mindfulness by comprehensively examining mindfulness-driven resting state functional connectivity (rsFC) outcomes.

### The neurocognitive features of mind wandering (MW)

When measured as functional connectivity, changes in neural circuitry reflect strengthened or weakened coordination between regions and, at a larger scale, between networks of interest^38,39^. Analogous to the spontaneous flow of thought characteristic of resting states, the brain’s activity at rest self-organizes into temporally-coherent neural networks detected by blood oxygen level dependent (BOLD) signal^40,41^. Such networks are commonly termed ‘resting-state’ or ‘intrinsic’ functional networks^42^. Although there is little consistency in the taxonomy of intrinsic functional networks (for review see Ref.^43^), it is generally accepted that a minimum of 10 networks are observable during the resting state^44^.

Among recognized large-scale networks, the DMN, SN, and FPCN have been frequently implicated in explanatory frameworks of MW. The DMN has received the most attention historically for its role in internally-directed mentation^17,45^. Indeed, prevailing evidence suggests that the primary function of the DMN may be the generation and maintenance of internally-oriented mental processes^46,47^. The DMN may be further parceled into three subnetworks, the midline-located core DMN, the medial temporal lobe subsystem (DMN-MTL), and the dorsomedial prefrontal cortex subsystem (DMN-dmPFC)^48^. While each subnetwork supports different functions of internally oriented mentation, the core DMN—encompassing the posterior cingulate cortex (PCC) and ventromedial prefrontal cortex (vmPFC)—is most robustly associated with self-referential thought^48–51^. Hyperconnectivity within the core DMN has been reliably observed in psychopathologies characterized by self-focused perseverative cognition (i.e., depression)^52–54^; however, the role of the DMN in maladaptive MW is likely more complex. In support of this viewpoint, neural models of MW suggest that the regulation (or dysregulation) of internally-oriented mental states relies on inter-network coordination between the DMN and networks implicated in attention, namely the SN and FPCN^37,55,56^.

According to the dynamic framework of mind-wandering^4^, internal experiences—maintained by the DMN—may be deliberately or automatically constrained via coordination with the FPCN and SN, respectively. The FPCN, characterized by dorsolateral PFC and anterior inferior parietal structures^43^, is well-known for its integral role in cognitive control processes^57^. Studies combining experience sampling with neuroimaging suggest that the FPCN flexibly couples with the DMN to deliberately regulate internal mentation^58^, and that such coordination is used to inhibit internal thought when external attention is required^59,60^.

In contrast, the SN has been theorized to support automatic constraints on internal experiences through cognitively efficient cross-network signaling^4^. Key nodes of the SN include the dorsal anterior cingulate cortex (dACC)^61^ and anterior insula^43^, which jointly operate within a cortico-striato-thalamo-cortical loop to detect motivationally important stimuli, either internal or external^62^. Through its extensive connectivity with the DMN and FPCN, the SN facilitates “set-shifting” by automatically and flexibly directing attention to and from internal and external cues^55,63^. Interestingly, SN engagement has also been suggested as a putative mechanism of perseverative cognition^64^. According to this viewpoint, aberrant SN connectivity exacerbates interoceptive awareness of unpleasant sensations, and due to the high motivational value of ruminative thoughts, draws attentional resources to internally-directed mentation^64^. While this theory is indirectly supported by evidence of SN hyperconnectivity in high-rumination individuals^65,66^, it fails to explain why therapies aiming to enhance awareness of internal experiences (i.e., mindfulness) would ameliorate ruminative thought. Thus further research is warranted to elucidate how cognitive training, like mindfulness, alters the dynamics of internal experiences at both a subjective and neurobiological level.

Multi-method investigations suggest that variability in functional connectivity within and between networks likely reflects the content and dynamics of internal mentation^6,67,68^, and resting state functional connectivity (rsFC) has been associated with individual differences in symptoms of anxiety^69^, depression^52,70^, and trait rumination^71,72^. Thus reorganization of resting state neurocircuitry, as detected by rsFC protocols, has been suggested as a key mechanism of treatment for psychopathologies featuring repetitive negative thought^73–77^. Although the research is nascent, there is initial evidence that mindfulness training may improve regulation of mental states, and that rsFC indices may be used to better understand the mechanisms through which mindfulness extends its therapeutic effects (e.g., Ref.^78^).

### Mechanisms of mindfulness: theory and empirical support

Mindfulness, commonly defined as the act of attending to present-moment thoughts, emotions, and sensations without judgment or appraisal^79^, is relatively unique as a treatment of maladaptive MW. Unlike popular cognitive behavioral therapies (CBTs), which enable the individual to challenge dysfunctional thoughts^80^, mindfulness instead targets one’s relationship to such thoughts^81,82^. This non-judgmental stance towards internal experiences may be promoted through a combination of neurocognitive mechanisms (for review see Refs.^24,29,30,83–86^). Mounting evidence indicates that mindfulness practice enhances *attentional control*, which may potentially support the deliberate constraint of maladaptive MW^64^. Foundational to the practice of mindfulness is the development of attentional control through a meditative technique called Focused Attention (FA). FA meditation trains the practitioner to focus on and maintain attention to a neutral sensory object (e.g., the breath), and direct attention back to that object when the mind begins to wander^87^. This recursive process of shifting and sustaining attention has previously been linked to enhanced functional connectivity within the FPCN of experienced meditators^23^, suggesting that FA improves top-down cognitive control needed to disengage from distracting thoughts and emotions.

Alternative models of mindfulness posit that mindfulness may *indirectly* regulate MW by promoting *awareness* of internal experiences^85^. According to this framework, the sustained concentration conferred by mindfulness facilitates awareness (or mindful meta-awareness), defined as the capacity to observe one’s mental patterns with a sense of equanimity and psychological distance^87,88^. This internal awareness thereby supports recognition of thoughts and feelings as discrete mental states, and in turn, improves flexible, adaptive responding^87^. The cultivation of mindful awareness has been theoretically attributed to enhanced functional cohesion of networks linked to self-awareness (e.g., default mode network) and attention monitoring (e.g., salience networks)^23,30^. However, support for this neural model draws largely from cross-sectional research (e.g., Refs.^23,89,90^), as well as correlational evidence^33–36^. Moreover, this neural model of mindful awareness does not fully account for the role of *non-judgment, or acceptance*, which may operate in tandem with awareness to reduce “experiential fusion” with one’s thoughts^91,92^. It has been speculated that mindfulness may dampen experiential fusion through DMN downregulation^85,92^; however, it is unclear how neural substrates of attention and awareness may mediate such effects.

Building on previous models of mindfulness meditation^24,29,30,85^, it is plausible that mindfulness training (MT) alters the resting state via reorganization of neural circuitry (i.e., intrinsic functional connectivity). Specifically, we posit that focused attention training recruits the FPCN, implicated in cognitive control and executive function. Given its documented role as a ‘hub’ of functional connectivity^58^, the FPCN may plausibly facilitate functional connections between other resting state networks—particularly between networks related to mind-wandering (i.e., default mode network) and internal awareness (i.e., salience network)—thus enabling the flexible regulation of mental states^85^. Coupled with improvements in executive functioning, such functional coordination between DMN and SN may likewise support meta-awareness skills needed to identify and disengage from maladaptive cognitive patterns. To extend this theoretical framework, the present study posed the question: Does mindfulness training alter functional connectivity between and within DMN, salience, and FPCN during rest?

### Present study

The aim of this study was to determine if mindfulness training modifies intrinsic functional connectivity (IFC) observed during resting states. Specifically, this study sought to examine connectivity within and between the frontoparietal control network (FPCN), the default mode network (DMN), and the salience network (SN). To date, several studies have investigated the impact of mindfulness training on resting state functional connectivity (rsFC) using controlled, experimental designs^78,89,93,94^. Although insightful, such studies typically suffer from low statistical power, a limitation endemic to research relying on high-cost neuroimaging modalities such as fMRI. Addressing such concerns, meta-analytic approaches may be used to pool information from well-controlled studies while modeling convergence of effects across pooled samples. Thus, we conducted a systematic review and meta-analysis of rsFC outcomes of mindfulness training relative to structurally-equivalent programs (i.e., active controls). To test the neuroplastic changes associated with mindfulness skills—namely, executive functioning and meta-awareness—we hypothesized that (1) mindfulness training would enhance rsFC between the FPCN and DMN as an indicator of enhanced cognitive control; (2) mindfulness training would enhance rsFC between the DMN and SN, reflective of meta-awareness; and (3) mindfulness training would alter rsFC within such networks.

## Results

Study characteristics and participant demographics are displayed in Table 1. Overall, studies used standardized mindfulness-based interventions ranging from 3 days to 8 weeks in length. All studies except for one featured designs with a structurally equivalent control intervention. Study samples varied in terms of age (*M* = 45.80; *SD =* 13.15) and clinical characteristics with the majority of studies recruiting healthy adults (see Table 1). The majority of individuals from the pooled sample identified as female (*n* = 286, 62.72%) followed by male (*n* = 170, 37.28%). No studies reported data from trans or non-binary participants. Of the 12 included studies, only 5 reported racial/ethnic demographic information. From this subsample of 152 participants, 100 (65.79%) identified as white, 31 (20.39%) as Black/African American, 12 (7.89%) as Mixed Race/Other, 5 as Hispanic/Latino (3.29%), 3 (1.97%) as Asian, and 1 as Southeast Asian (0.66%).

**Table 1:** Demographic characteristics of mindfulness and control conditions.

Reference	Population description	Program description		*n* analyzed		Age *M* (SD)		Biological sex *n* (%)	
		MT	Control	MT	Control	MT	Control	MT	Control
Brewer et al.^89^	Individuals with > 10 year mindfulness meditation experience vs. meditation-naive controls	NA—quasi-experimental design		*n* = 13	*n* = 12	51.5 (6.8)	49.4 (6.2)	*Male* 5 (41.7%)	*Male* 6 (50%)
								*Female* 7 (58.3%)	*Female* 6 (50%)
Chumachenko et al.^144^	Individuals who recently lost weight intentionally and were engaged in weight loss maintenance	Mindfulness Based Stress Reduction (MBSR)	Structurally equivalent Healthy Living Course	*n* = 28	*n* = 23	44.5 (9.70)	44.5 (10.65)	*Male* 7 (24%)	*Male* 4 (13%)
								*Female* 22 (76%)	*Female* 24 (87%)
Creswell et al.^78^	Stressed unemployed community adults	3-day mindfulness meditation retreat	Structurally equivalent relaxation training intervention	*n* = 17	*n* = 17	37.94 (10.96)	41.00 (9.55)	*Male* 11 (61.11%)	*Male* 9 (52.94%)
								*Female* 7 (38.89%)	*Female* 8 (47.06%)
King et al.^94^	Male combat veterans with diagnosed PTSD	16-week nontrauma-focused mindfulness-based exposure therapy; incorporates elements from MBCT and PTSD psychoeducation	16-week present-centered group therapy; controls for nonspecific therapeutic factors	*n* = *12*	*n* = *8*	32.43 (7.54)	31.67 (10.14)	*Male* 14 (100%)	*Male* 9 (100%)
								*Female* 0 (0%)	*Female* 0 (0%)
Kral et al.^32^	Healthy meditation-naive adults	MBSR	Structurally equivalent Health Enhancement Program (HEP)	*n* = *31*	*n* = *34*	41.4 (12.9)	43.6 (13.1)	*Male* 13 (41.94%)	*Male* 12 (35.29%)
								*Female* 18 (58.06%)	*Female* 22 (64.71%)
Kwak et al.^141^	Healthy office workers and graduate students	3-day mindfulness meditation retreat at a Buddhist temple	3-day relaxation retreat without structured activities	*n* = *30*	*n* = *17*	30.63 (4.97)	31.71 (5.02)	*Male* 6 (20%)	*Male* 5 (29.41%)
								*Female* 24 (80%)	*Female* 12 (70.59%)
Rahrig et al.^145^	Stressed, meditation-naive community adults	2-week remote delivered mindfulness training	Structurally equivalent training in active coping techniques	*n* = 11	*n* = 12	33.36 (7.30)	35.67 (8.54)	*Male* 2 (18%)	*Male* 6 (50%)
								*Female* 9 (82%)	*Female* 6 (50%)
Shao et al.^140^	Healthy elderly adults with no prior meditation or relaxation training experience	8-week attention-based compassion meditation training	Structurally equivalent relaxation training	*n* = *21*	*n* = *19*	64.78 (2.71)	64.68 (2.19)	*Male* 7 (30%)	*Male* 8 (36%)
								*Female* 16 (70%)	*Female* 14 (64%)
Taren et al.^138,139^	Stressed unemployed job-seeking community adults	3-day mindfulness meditation retreat	Structurally equivalent relaxation training intervention	*n* = *17*	*n* = *17*	37.94 (10.96)	41.00 (9.55)	*Male* 11 (61.11%)	*Male* 9 (52.94%)
								*Female* 7 (38.89%)	*Female* 8 (47.06%)
Turpyn et al.^142^	Stressed mothers of adolescent children	8-week parenting focused mindfulness intervention based on MBSR	Structurally equivalent parenting education intervention	*n* = *10*	*n* = *10*	*Combined Programs* 48.5 (7.62)		*Male* 0 (0%)	*Male* 0 (0%)
								*Female* 10 (100%)	*Female* 10 (100%)
Van der Gught et al.^143^	Breast cancer survivors reporting cognitive impairment	8-week mindfulness-based intervention developed for patients with cancer	Waitlist controlled condition	*n* = *12*	*n* = *13*	43.89 (6.03)	47.4 (5.45)	*Male* 0 (0%)	*Male* 0 (0%)
								*Female* 18 (100%)	*Female* 15 (100%)
Wells et al.^137^	Adults with mild cognitive impairment	MBSR	Care as usual	*n* = *8*	*n* = *5*	73 (8.00)	75 (7.00)	*Male* 3 (33%)	*Male* 6 (60%)
								*Female* 6 (67%)	*Female* 2 (40%)

Seed regions were pooled according to standardized resting state network location. This process demonstrated that eligible studies used seed regions from four networks, the default mode network (DMN; *n* = 11), the midcingulo-insular network (M-CIN; *n* = 7), the dorsal attention network (DAN; *n* = 1), and the frontoparietal control network (FPCN; *n* = 2) (see Supplementary Table S1).

**Figure 1:**
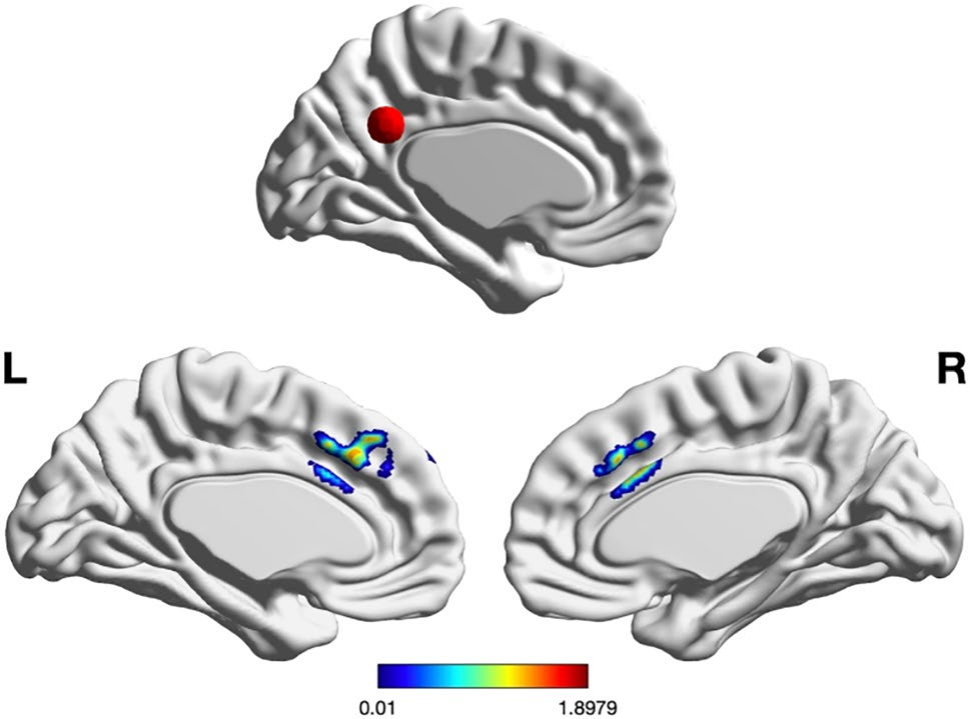
The top figure portrays the location of the posterior cingulate cortex (PCC) ROI seed. The bottom figures show a 3D map of voxelwise z-scores, with significant cluster effects localized to the left dorsal anterior cingulate cortex (dACC) (BA 24).

**Figure 2:**
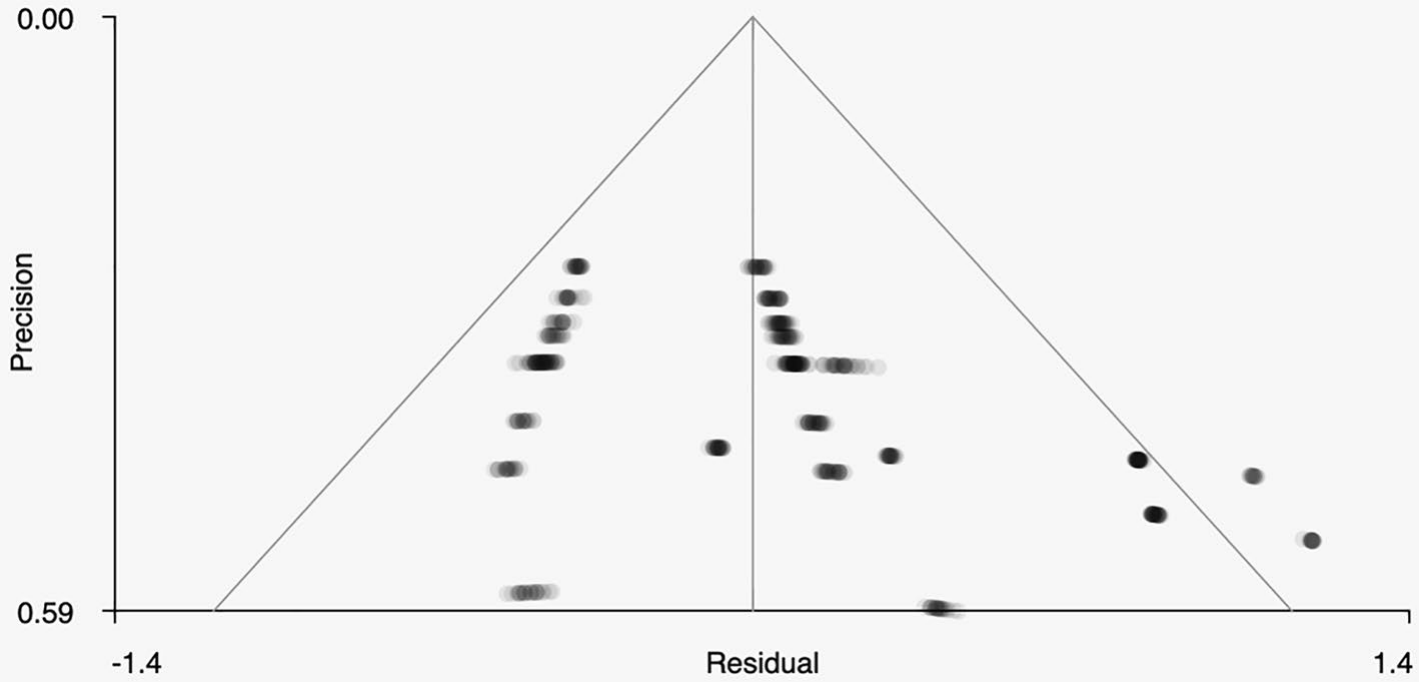
Funnel plot indicating relative symmetry in the scatter of studies based on effect index, i.e., residual (x-axis) and sample size index, i.e., precision (y-axis). Plot symmetry suggests the absence of publication bias due to trial size.

SDM meta-analysis was used to test for significant training condition effects. Meta-analysis results identified one significant cluster of 57 voxels loc ated in the paracingulate gyri (Table 2, Fig. 1), *Hedge’s g* = 0.234, *uncorrected p* = 0.0288, *I*^2^ = 15.87, suggesting that mindfulness training, relative to control training programs, increased connectivity to bilateral paracingulate gyri and the left anterior cingulate (non-significant cluster outcomes reported in Supplementary Table S2). Yeo’s cortical parcellation atlas^147^ places the peak coordinates of this cluster (0, 20, 34) in the ventral attention network (VAN), more recently taxonomized as the mid-cingulo insular network (within the anatomical domain) or salience network (within the cognitive domain)^47^. Egger’s test for publication bias was nonsignificant, bias = 2.17, *p* = 0.162, and funnel plots did not suggest the influence of small study effects (Fig. 2).

**Table 2:** Significant Clusters Identified from Meta-analysis.

MNI coordinate	SDM-Z	*P*	Description
0, 20, 34	1.898	0.028856218	Left median cingulate/paracingulate gyri, BA 24

Brain region	Voxels		
Left median cingulate/paracingulate gyri, BA 24	13		
Right median cingulate/paracingulate gyri, BA 24	12		
Left median cingulate/paracingulate gyri	2		
Left anterior cingulate/paracingulate gyri, BA 24	2		
Right median cingulate/paracingulate gyri	1

**Figure 3:**
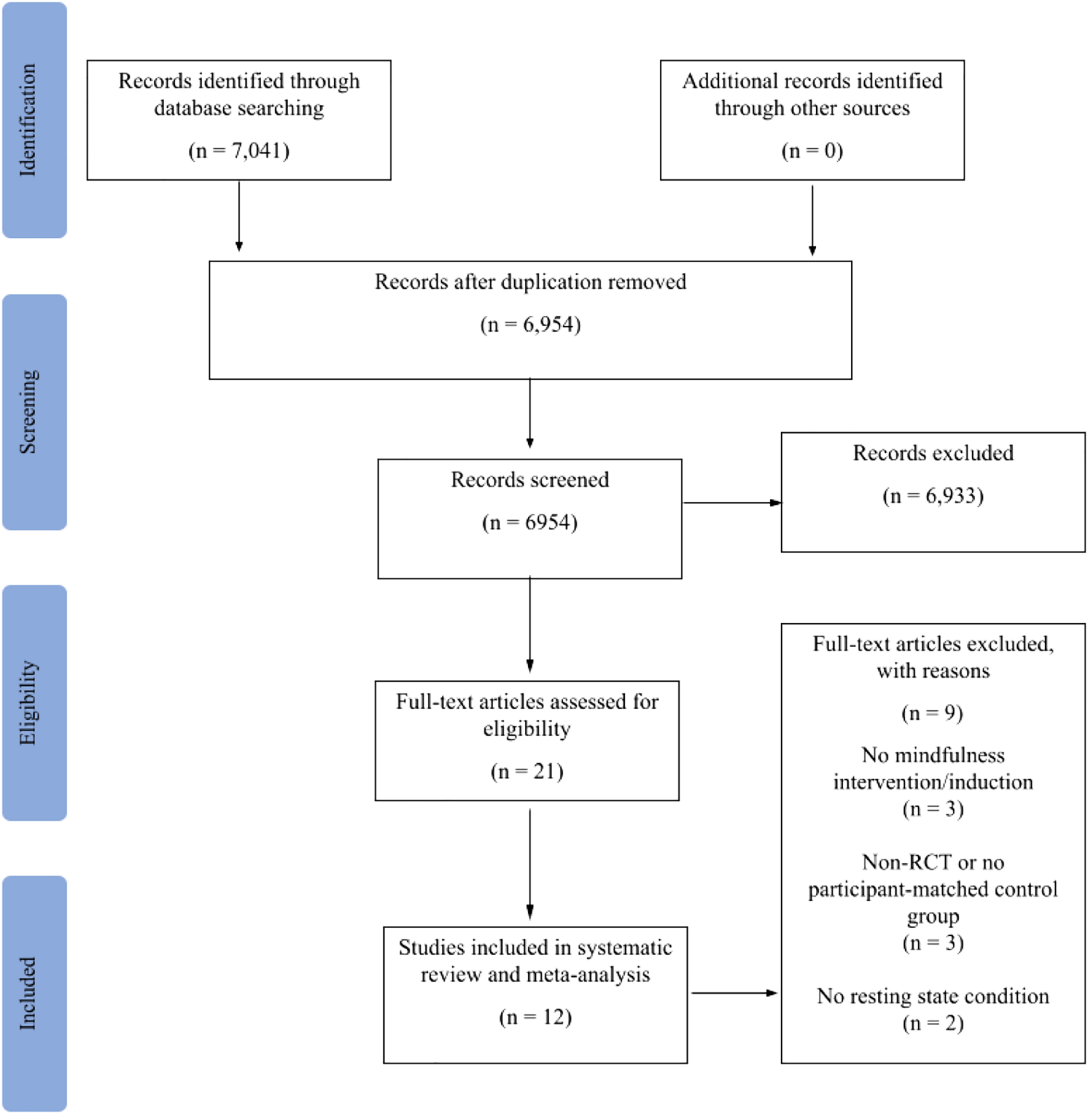
PRISMA flow diagram depicts records identified and screened for eligibility for the meta-analysis.

Examining the original articles revealed increased functional connectivity to the median cingulate via the posterior cingulate cortex (PCC), a region canonically situated within the default mode network (DMN). Notably, studies within the sample exclusively reported increased connectivity between median cingulate effect regions and PCC seed regions. Therefore, the findings suggest that mindfulness training increased resting state functional connectivity between the default mode network and salience network.

## Discussion

This meta-analysis is the first to systematically examine the effects of mindfulness-based training on resting state functional connectivity (rsFC), a neural marker of cognitive and emotion regulation. A systematic review of the literature revealed that rsFC has been sparsely investigated as a target of mindfulness training, with 12 studies meeting the eligibility criterion in the current review. Meta-analysis results partially supported our hypotheses, indicating that relative to mindfulness naive participants and participants trained in one or another structurally equivalent program, mindfulness trainees increased functional connectivity to the dorsal anterior cingulate cortex (dACC), and that such connectivity was seeded to the posterior cingulate cortex (PCC). Results did not support our first hypothesis, which predicted enhanced cross-network connectivity between the FPCN and DMN as a mechanism of cognitive control. In support of our second hypothesis, these results suggest that mindfulness training strengthened cross-network connectivity between regions associated with the default mode network (DMN) and salience network (SN). Implications for supported and null findings are explored in the following paragraphs.

Along with the frontoparietal control network (FPCN), the DMN and SN operate synergistically to support self-regulation, and aberrant coordination within and between these networks has been associated with emotional dysfunction, deficits in attentional control, and maladaptive mind wandering (MW) (e.g., rumination, worry, intrusive thought)^78,95^. It has been suggested that therapeutic outcomes of mindfulness are mediated by reorganization within and between such networks^24,29,30,85^; however, the precise neural targets of mindfulness are ill-defined, in no small part due to methodological differences between studies (e.g., in research designs, training protocols, and target populations)^96^. Among explanations of mindfulness’ neurocognitive mechanisms, the literature favors two competing theories, colloquially referred to as ‘top-down’ and ‘bottom-up’ models of mindful regulation. According to the top-down model, mindfulness recruits FPCN engagement to support the deliberate regulation of unwanted thoughts and emotions^30,97^, and—through dynamic cross-network coupling—inhibits task-incongruent DMN activity^58^. In contrast, bottom-up models suggest that mindfulness can operate automatically to modify thoughts and emotions without higher-level cognitive control^98^. Specifically, such bottom-up regulation may diminish the propensity for rumination by reducing within-network DMN connectivity^99,100^ or disrupting SN-DMN coordination associated with negative rumination^64^. We suggest that the effect of mindfulness training on dACC-PCC connectivity may partially align with models of both top-down and bottom-up regulation.

Contrary to our prediction, this meta-analysis did not reveal involvement of FPCN cross-network connectivity, a finding that is inconsistent with prior neural characterizations of long-term meditators^101^ and runs counter to theoretical explanations of the FPCN as a facilitator of mindful top-down regulation^30,97^. On the one hand, this null effect may derive from inadequate statistical power, given that only 2 of the included studies investigated FPCN-seeded rsFC. On the other hand, such findings do not necessarily rule out mechanisms of top-down cognitive control. It has previously been suggested that executive control processes may operate via dissociable networks with distinct temporal profiles^64,102^. According to this framework, the FPCN provides rapid, flexible feedback to lower-level systems, while the cingulo-opercular network (analogous to the salience network^43^) functions to maintain cognitive control over longer epochs^102^. Included within the cingulo-opercular network is the dACC, the functional connectivity target region identified from our analysis. Otherwise referred to as the midcingulate cortex^61^, the dACC is distinguished by specialty Von Economo neurons^103,104^. Von Economo neurons—or spindle neurons—are characterized by long-distance signal transmission and heterogenous dendritic/spinal structures^105,106^, two features which appear to support cross-network information integration^103,104,107,108^. Such extensive connectivity supports global monitoring of internal and external experiences^109^, and by extension, evaluates the degree of cognitive effort needed for a given situation^110^. In the early stages of mindfulness practice, when attentional focus is described as “effortful”^30^, dACC-PCC coupling may reflect the deliberate regulation of sympathetic arousal to maintain a state of alert focus. Such an explanation is plausible given that the studys’ participants included in this analysis were exposed to relatively brief training durations, with the majority ranging between 3 days to 8 weeks. It is possible that continued practice may induce neuroplastic changes indicative of “effortless” attention regulation as exhibited by long-term or expert practitioners^109^.

Alternatively, increased PCC-dACC functional connectivity may support indirect (i.e., bottom-up) regulation through the promotion of meta-awareness. Embedded within the DMN, the PCC has classically been associated with the maintenance of internally-oriented mentation^17^. However, the PCC may also play a critical role in attention regulation^111^ and the general maintenance of vigilance^112,113^. Evidence suggests that dorsal portions of the PCC may be involved in balancing internal and external attentional focus^56^ as facilitated through extensive cross-network connectivity^111,114^. In this vein, PCC connectivity with the dACC—a salience network hub—may serve to integrate information regarding the content, quality, and direction of attentional focus (i.e., meta-awareness). Thus, our finding of dACC-PCC connectivity may potentially underpin flexible cognitive faculties needed to observe internal states with open awareness.

Interpretation of the results reported here is limited by several factors. To date very few studies have examined rsFC outcomes of mindfulness, with even fewer implementing randomized controlled or quasi-experimental procedures. For this reason the meta-analysis pulls from a relatively small sample of 12 studies (MT *n* = 226; CT *n* = 224). Although such selectivity maintains the benefits of data quality and homogeneity of experimental conditions (i.e., mindfulness interventions), small samples also threaten generalizability as effects may be driven by only a few studies with shared experimental parameters^115^. The extracted studies draw from heterogeneous populations—in terms of demographic characteristics, gender, and age—features which have shown to vary in both gray matter and connectome characteristics^116–118^. Additionally, no study reported non-binary gender information and reports of racial/ethnic identity were omitted from 7 of the 12 studies included in our sample. The practice of demographic underreporting is a substantial challenge in the field of mindfulness research (and psychological research more broadly), with important implications for health inequities^119^. Future researchers should prioritize responsible reporting and recruitment practices to address such disparities^119,120^.

The duration and therapeutic focus of mindfulness interventions likewise warrant consideration. Although the majority of mindfulness interventions were standardized, these studies report a wide range of intervention durations (3 days to 16 weeks) with different degrees of intensity (e.g., retreat verses remote-delivered trainings) and therapeutic focus (e.g., PTSD, parent-focused stress reduction). There is currently little research examining the dose–response relation for mindfulness-based treatments; however, a recent meta-analysis of this topic suggests that brief trainings may be equally as effective as longer interventions for the treatment of stress, depression, and anxiety^121^. Nevertheless, the relation between mindfulness training dose and neuroplasticity remains poorly understood, a matter that is further complicated when applied to clinical and aging populations with atypical connectomes^121–123^.

Interpretation is likewise limited by a priori seed selection, which was requisite for all included studies (see protocol from Ref.^52^). This meta-analysis failed to detect significant FPCN cross-network connectivity, and while this null effect may stem from erroneous theoretical assumptions, it may instead be the consequence of preferencing seeds within the DMN. Behavioral neuroimaging studies have previously shown strong functional coupling between the FPCN and DMN across multiple tasks (e.g., Refs.^59,124,125^, and one study recently demonstrated how robust co-activation of FPCN and DMN regions may obscure detection of group effects^126^. Further research is needed to definitely determine if and how mindfulness training interacts with FPCN neurocircuitry during task-related and resting brain states.

Finally, it warrants noting that there is currently no standard classification system for large-scale functional networks. The lack of universal nomenclature presents a significant barrier to interpreting neural outcomes, especially given the multitude of naming schemes^43^ and inconsistent application of common labels (see meta-analysis of executive control network topography^127^). We use the functional network terms “frontoparietal control network”, “default mode network”, and “salience network” primarily due to their ubiquity in cognitive neuroscience^43^ and the mindfulness literature. Nevertheless, such naming conventions based on functional properties are problematic for the reasons described above. We recommend that future research move towards more transparent network taxonomies incorporating anatomical properties (see Ref.^43^).

This meta-analysis highlights the potential value of rsFC as a window into understanding the mechanisms of mindfulness. However, research on this topic is in its early stages, and considerably more research is necessary to qualify specific rsFC effects as mechanistic targets of mindfulness training. Without additional research, the effects of mindfulness on resting state cognition remains speculative. Researchers may consider novel sampling methods including lab-based phenomenological reporting (e.g., Ref.^128^) and ecological momentary assessment, a method used to capture day-to-day lived experiences^129,130^. Investigators should additionally consider examining multiple representations of connectivity—including effective connectivity, white matter connectivity, and dynamic connectivity—which probe different features of brain network organization (e.g., directional effects; temporal dynamics, etc.). The comparison of such representations has the potential to reduce ambiguity and improve interpretation of connectivity-based effects^131^.

Further research is also needed to determine the relevance of rsFC plasticity for different psychiatric conditions. The successful use of network neuroscience for clinical diagnosis and treatment is a lofty goal, which will require overcoming considerable methodological challenges (see Refs.^132,133^). Such research necessitates reliability and reproducibility; however, preprocessing and analysis procedures may vary significantly among studies based on individual research questions. Nevertheless, researchers can promote standardization of analysis techniques through commitment to open science practices in which scripted pipelines and nonthreshold brain images are publicly catalogued (for recommendations, see Ref.^131^).

## Conclusion

Resting state neural indices have potential to elucidate the rich subtleties of internal experiences, with important implications for those suffering from rigid or negative inner dialogues. According to Buddhist perspectives, mindfulness offers a window into exploring the qualities of conscious experience and—through heightened awareness of such mental states—enables their adaptive transformation^84^. However, it remains ambiguous how mindfulness practice can be optimized to reduce suffering in heterogeneous populations, both healthy and clinical^119,134,135^. The current meta-analysis aimed to elucidate the nature of mindfulness training effects by focusing on neural indices of the resting mind. Results indicated strengthened dACC-PCC connectivity as a product of mindfulness. Although interpretation of this effect requires more experimental research, results may nevertheless advance the science of mindfulness and its impact on the resting brain.

## Methods

### Literature search

A comprehensive literature search was conducted using the keywords rest*(-ing), connect*(-ivity), *default mode*, *mindfulness*, *meditation*, *MBSR* [Mindfulness-based stress reduction], and *MBCT* [Mindfulness-based cognitive therapy] to search for matching literature from the following databases: MEDLINE/PubMed, ERIC, PSYCINFO, ProQuest, Scopus, and Web of Sciences (see Supplementary Table S4). Texts were screened by four reviewers and considered for inclusion if they reported resting state functional connectivity outcomes derived from functional magnetic resonance imaging (fMRI) modalities. Further, all included studies were randomized controlled trials or used quasi-experimental (e.g., matched control) designs in which mindfulness-based training programs (as operationally defined by Ref.^136^) were compared to a control condition.

Studies were excluded if they met one or more of the following criteria: (1) experimental interventions predominantly featured training elements other than mindfulness meditation (e.g., yoga, transcendental meditation, loving-kindness or compassion meditation, tai chi; integrative body-mind training); (2) resting state functional connectivity (rsFC) group contrasts were not reported; (3) seed-based rsFC methods were not used or seed-based rsFC indices were not reported.

The systematic literature review identified 7041 records, from which 6093 were excluded upon abstract review (see PRISMA flow diagram in Fig. 3). Full-text examination of the remaining 21 studies for eligibility yielded a sample of 13 eligible publications from 12 unique studies^32,78,89,94,137–145^. The final sample of studies reported data from 226 participants assigned to mindfulness training and 204 participants assigned to an active control program.

### Data extraction and coding

The meta-analysis was coordinate-based (for example see Ref.^52^), in which extracted coordinates reflected locations of significant group differences in resting state functional connectivity pre-post meditative training. Given that all studies used seed-based rsFC analyses, coordinates were categorized as belonging to either seed anatomy or effect anatomy. Using this coding scheme, 11 sets of seed coordinates were extracted, with coordinates reflecting each seed anatomy’s reported center of mass. In the instance that seed anatomy coordinates were not reported because the seed region was defined from a standardized atlas or subject-specific spatial map, the center of mass was estimated using meta-analytic maxima reported from the open source platform, neurosynth.org^146^. All studies reported peak coordinates of significant between-group effects, yielding a total of 65 effect coordinates. After extracting seed and effect coordinates, all coordinates were categorized into rsFC networks as defined from a standardized network cortical parcellation atlas^147^. Effects were likewise characterized by direction of effect with positive effects reflecting relatively stronger functional connectivity in the mindfulness group (MT > CT), and negative effects indicating relatively stronger functional connectivity in the control group (CT > MT). In addition to main effects, demographic data and intervention characteristics were extracted for review. Effects were extracted by four independent reviewers who collected data from independent reports.

### Interrater reliability

We conducted a two-way random-effects ICC modeled with absolute agreement. Specifically, we tested for significant effects of rater ID on each quantitative measure. ICC estimates were within acceptable range (ICC > 0.6; *p* < 0.005), indicating a high degree of rater agreement. Next, unweighted Cohen’s kappa was calculated to examine reliability of extracted categorical variables. Results indicated substantial agreement^148,149^ between the raters’ judgements, *k* = 0.741 (95% CI 0.182 to 0.884). Finally, z statistics were converted to two-sample t-test statistics, assuming equal variances in both conditions. If records did not report *z* statistics or two-sample *t*-test statistics, *p* values were used to estimate *t*-test statistics used in the meta-analysis.

### Voxel-based meta-analysis

While standard meta-analytic procedures require 3D statistical parametric maps, such images are often inaccessible in published fMRI reports. This limitation has prompted the development of coordinate-based meta-analytic (CBMA) approaches, which only require peak coordinates of significant clusters rather than 3D statistical images^150^. Nevertheless, CBMA procedures assume that voxels are independent and that the likelihood of false positives is equivalent among voxels^151^. Such assumptions may be overcome through voxel-based meta-analytic procedures, namely seed-based *d* mapping (SDM) with permutation of subject images (PSI)^151^. Unlike other (CBMA) approaches—namely, Activation Likelihood Estimation (ALE)^152^ and Multilevel Kernel Density Analysis (MKDA)^153^—which test for the presence or absence of peaks of statistical significance (i.e., null hypothesis testing), seed-based *d* mapping (SDM) uses multiple imputation to model effect sizes for each study before conducting a random-effects meta-analysis. By imputing effect sizes on 3D statistical maps, SDM has the advantage of both parametric mapping and coordinate-based approaches while controlling for familywise error rate (FWER)^154^ via threshold-free cluster enhancement (TFCE)^155^.

Thus, the current meta-analysis was conducted using SDM (SDM-*PSI* version 6.21) with permutation of subject images (PSI) CBMA algorithm^156^. SDM preprocessing was first used to convert *t*-values of peak coordinates into Hedge’s *g* effect sizes. Study-level images of upper and lower bounds of probable effects were then constructed for all voxels using multiple imputation^156,157^, in which anisotropic Gaussian kernels are convolved with reported effect sizes^158^. Using SDM we then calculated most likely effect size and standard error via multiple imputations of Maximum Likelihood Estimation (MLE) with a jackknife procedure. Finally, FWE corrections were performed via subject-based permutation testing and TFCE-corrected effect sizes were calculated to estimate group differences. Main analytic findings were scrutinized for small study effects and excess significance (i.e., publication bias) through Egger’s tests and examination of funnel plots. Finally, given that atypical functional connectivity is a transdiagnostic feature of pathological populations^118^, we conducted a meta-regression using random-effects general linear modeling to explore the potential confounding effect of clinical population status. Null effects are reported in Supplementary Table S3.

## Supplementary Information

Supplementary Table S1.

Supplementary Table S2.

Supplementary Table S3.

Supplementary Table S4.

## Data Availability

The datasets generated during and/or analysed during the current study are available in the neurovault repository, https://identifiers.org/neurovault.image:768613. Review protocol is not registered and can only be accessed upon request.
