# Supplementary material for: Meta-analytic evidence that mindfulness training alters resting state default mode network connectivity
Source: Sci Rep. 2022 Jul 18;12:12260. doi: 10.1038/s41598-022-15195-6 (PMC9293892; doi:10.1038/s41598-022-15195-6)
Supplement: Supplementary file 1 — Supplementary Table S1. [file 41598_2022_15195_MOESM1_ESM.docx]

**Supplemental Table 1.**

| Reference | Direction | Seed network | Seed Anatomy (MNI) | Effect network | Effect anatomy (MNI) |
| --- | --- | --- | --- | --- | --- |
| **Brewer et al. (2011)** | MT > CT | Default Mode | left medial prefrontal cortex (-6, 52,-2) | Limbic | left precuneus (-24, -3, -36) |
|  |  |  |  | Dorsal attention | left fusiform/inferior temporal gyrus (-48, -54, -21) |
|  |  |  |  | Ventral attention | right inferior parietal lobule (63, -24, 42) |
|  |  |  |  | Frontoparietal | right cerebellum/inferior occipital (27, -69, -24) |
|  | MT > CT | Default Mode | left posterior cingulate cortex (-8, -56, 26) | Default Mode | right middle/superior temporal gyrus (60, -39, -3) |
|  |  |  |  | Limbic | rostral superior frontal gyrus (-27, 60, -12) |
|  |  |  |  | Frontoparietal | left middle/inferior frontal gyrus (-48, 36, 15) |
|  |  |  |  | Somatosensory | left superior temporal/postcentral gyrus/insula (-51,-18, 9) |
|  |  |  |  | Dorsal attention | inferior temporal/fusiform gyrus (-51, -60, -15) |
|  |  |  |  | Ventral attention | dorsal anterior cingulate gyrus (0, 6, 30) |
|  |  |  |  | Visual | left posterior cingulate (-18, -66, 6) |
|  |  |  |  | Visual | left middle occipital gyrus (-45, -87, 21) |
|  |  |  |  | Ventral attention | right postcentral gyrus/inferior parietal lobule (72, -21, 27) |
|  |  |  |  | Dorsal attention | left precuneus (-24, -66, 27) |
|  | CT > MT | Default Mode | left posterior cingulate cortex (-8, -56, 26) | Frontoparietal | right dorsal caudate (12, -24, 27 ) |
|  |  |  |  |  |  |
| **Chumachenko et al. (2021)** | MT > CT | Midcingulo-insular | left amygdala (-22, 0, -20) | Default | left vmPFC (-2, 44, -8) |
| MT n = 29 | MT > CT | Midcingulo-insular | right amygdala (24, 0, -20) | Default | right vmPFC (2, 60, -8) |
| CT n = 23 |  |  |  |  |  |
|  |  |  |  |  |  |
| **Creswell et al. (2016)** | MT > CT | Default Mode | left posterior cingulate cortex (-4, -50, 40) | Visual | left lingual gyrus (-12, -78, -4) |
| MT n = 17 |  |  |  | -- | left pallidum (-20, -4, 2) |
| CT n = 17 |  |  |  | Frontoparietal | left middle frontal gyrus (-22, 52, 10) |
|  |  |  |  | Frontoparietal | left insula (-26, 18, -8) |
|  |  |  |  | Somatosensory | left thalamus (-28, -26, 8) |
|  |  |  |  | Ventral Attention | ventral attention (-30, 42, 38) |
|  |  |  |  | Default | left inferior frontal gyrus (-46, 32, 0) |
|  |  |  |  | Somatosensory | left precentral gyrus (-52, -12, 36) |
|  |  |  |  | -- | left pallidum (-6, 0, -6) |
|  |  |  |  | Somatosensory | right precentral gyrus (10, -6, 80) |
|  |  |  |  | Frontoparietal | right superior frontal gyrus (20, 62, -6) |
|  |  |  |  | Somatosensory | right insula (32, -26, 20) |
|  |  |  |  | Visual | right fusiform gyrus (36, -62, -14) |
|  |  |  |  | Somatosensory | right paracentral lobule (4, -34, 64) |
|  |  |  |  | Limbic | right inferior temporal cortex (46, 2, -44) |
|  |  |  |  | -- | right postcentral gyrus (48, -4, 28) |
|  |  |  |  | Frontoparietal | right supramarginal gyrus (60, -44, 38) |
|  |  |  |  |  |  |
| **King et al. (2016)** | MT > CT | Default Mode | posterior cingulate cortex (0, -56, 20) | Frontoparietal | left dorsal anterior cingulate cortex (-3, 23, 40) |
| MT n = 12 |  |  |  | Frontoparietal | left dorsolateral prefrontal cortex (-30, 47, 10) |
| CT n = 8 |  |  |  | Frontoparietal | left dorsolateral prefrontal cortex (-33, 47, 19) |
|  |  |  |  | Frontoparietal | left dorsal anterior cingulate cortex (-6, 32, 37) |
|  |  |  |  | Frontoparietal | right dorsolateral prefrontal cortex (36, 41, 16) |
|  |  |  |  |  |  |
| **Kral et al. (2019)** | MT > CT | Default Mode | left posterior cingulate cortex (-4, -50, 40) | Limbic | right inferior temporal gyrus (56, -40, -18) |
| MT n = 31 |  |  |  |  |  |
| CT n = 34 |  |  |  |  |  |
|  |  |  |  |  |  |
| **Kwak et al. (2019)** | MT > CT | Midcingulo-insular | tempo-parietal junction | Default | left dorsomedial prefrontal cortex (-10, 54, 36) |
| MT n = 30 |  |  |  | Default | left tempoparietal junction (-50, -56, 20) |
| CT n = 17 |  |  |  | Default | precuneus (0, -48, 28) |
|  |  |  |  | Dorsal attention | right tempoparietal junction (50, -56, 20) |
|  |  |  |  |  |  |
| **Shao et al. (2016)** | MT > CT | Default Mode | left posterior cingulate cortex (-5, -49, 40) | -- | brainstem/pons (0, -33, -30) |
| MT n = 21 | CT > MT | Default Mode | left posterior cingulate cortex (-5, -49, 40) | Somatosensory | right postcentral/precentral gyrus (4, 24, -39) |
| CT n = 19 |  |  |  |  |  |
|  |  |  |  |  |  |
| **Taren et al. (2015a)** | CT > MT | Midcingulo-insular | left amygdala | Limbic | subgenual anterior cingulate cortex (0, 18, -12) |
| MT n = 17 |  |  |  |  |  |
| CT n = 17 |  |  |  |  |  |
|  |  |  |  |  |  |
| **Taren et al. (2015b)** | MT > CT | Dorsal attention | left dorsolateral prefrontal cortex (-28, 0, 54) | Visual | left superior parietal lobule (-10, -78, 36) |
| MT n = 17 |  |  |  | Dorsal attention | left middle temporal/angular gyrus (-42, -58, 10) |
| CT n = 17 |  |  |  | Dorsal attention | right supplemental eye field (22, 12, 58) |
|  |  |  |  | Frontoparietal | right medial frontal gyrus (34, 2, 58) |
|  | MT > CT | Frontoparietal | left dorsolateral prefrontal cortex (-48, 36, 15) | Frontoparietal | right inferior frontal gyrus (54, 16, 14) |
|  | MT > CT | Frontoparietal | right dorsolateral prefrontal cortex (32, 50, 12) | Frontoparietal | right medial frontal gyrus (46, 20, 40) |
|  |  |  |  |  |  |
| **Turpyn et al. (2019)** | CT > MT | Midcingulo-insular | right posterior insula | Somatosensory | superior parietal lobule/precuneus (-16, -48, 66) |
| MT n = 10 | CT > MT | Midcingulo-insular | dorsal anterior cingulate cortex | Dorsal attention | precentral gyrus/middle frontal gyrus (-40, -4, 52) |
| CT n = 10 | CT > MT | Midcingulo-insular | right posterior insula | Dorsal attention | left posterior middle temporal cortex (-60, -60, 4) |
|  |  |  |  |  |  |
| **Van der Gught et al. (2020)** | MT > CT | Midcingulo-insular | anterior cingulate cortex (0, 22, 35) | Frontoparietal | left intraparietal sulcus (-39, -43, 52) |
| MT n = 12 |  |  |  | Dorsal attention | right intraparietal sulcus (39, -42, 54) |
| CT n = 13 |  |  |  |  |  |
|  |  |  |  |  |  |
| **Wells et al. (2013)** | MT > CT | Default Mode | right posterior cingulate cortex (8, -56, 30) | Default | bilateral superior medial prefrontal cortex (0, 50, 40) |
| MT n = 8 |  |  |  | -- | left hippocampus (28, -22, 16) |
| CT n = 5 |  |  |  | Default | bilateral orbital medial prefrontal cortex (4, 50, -10) |
|  |  |  |  |  |  |
| **Rahrig et al. (2021)** | MT > CT | Default Mode | posterior cingulate cortex (0, 50, -6) | Default | right ventromedial prefrontal cortex (4, 30, 18) |
| MT n = 10 |  |  |  |  |  |
| CT n = 12 |  |  |  |  | left precuneus (-24, -3, -36) |
|  |  |  |  |  |  |
|  |  |  |  |  |  |
|  |  |  |  |  |  |
|  |  |  |  |  |  |
|  |  |  |  |  |  |
|  |  |  |  |  |  |
