# Supplementary material for: Meta-analytic evidence that mindfulness training alters resting state default mode network connectivity
Source: Sci Rep. 2022 Jul 18;12:12260. doi: 10.1038/s41598-022-15195-6 (PMC9293892; doi:10.1038/s41598-022-15195-6)
Supplement: Supplementary file 2 — Supplementary Table S2. [file 41598_2022_15195_MOESM2_ESM.docx]

**Supplemental Table S2.**

| *MNI coordinate* | *SDM-Z* | *P* | Voxels | Description |
| --- | --- | --- | --- | --- |
|  |  |  |  |  |
| 0,20,34 | 1.898 | 0.028856218 | 384 | Left median cingulate / paracingulate gyri, BA 24 |
|  | | | | |
| 36,-68,-20 | 1.541 | 0.061705589 | 61 | Right fusiform gyrus, BA 19 |
|  | | | | |
| 60,-40,-12 | 1.556 | 0.059849858 | 31 | Right middle temporal gyrus, BA 20 |
|  | | | | |
| -48,36,6 | 1.429 | 0.076552451 | 22 | Left inferior frontal gyrus, triangular part, BA 45 |
|  | | | | |
| -8,52,36 | 1.377 | 0.084312439 | 4 | Corpus callosum |
|  | | | | |
| 48,20,22 | 1.353 | 0.088074028 | 4 | Right inferior frontal gyrus, triangular part, BA 48 |
|  | | | | |
| 32,-28,18 | 1.308 | 0.095488548 | 2 | Right superior longitudinal fasciculus III |
| *Note.* This table reports non-significant clusters (*p* < .1) identified from the seed-based d-mapping | | | | |
|  |  |  |  |  |
