# Supplementary material for: Meta-analytic evidence that mindfulness training alters resting state default mode network connectivity
Source: Sci Rep. 2022 Jul 18;12:12260. doi: 10.1038/s41598-022-15195-6 (PMC9293892; doi:10.1038/s41598-022-15195-6)
Supplement: Supplementary file 3 — Supplementary Table S3. [file 41598_2022_15195_MOESM3_ESM.docx]

**Supplemental Table S3. Meta-Regression Report**

| *MNI coordinate* | *SDM-Z* | *P* | Voxels | Description |
| --- | --- | --- | --- | --- |
|  |  |  |  |  |
| -4, 36, 40 | 1.492 | 0.067797601 | 27 | Left superior frontal gyrus, medial, BA 32 |
|  | | | | |
| 2, 34, 40 | 1.318 | 0.093708038 | 2 | Left superior frontal gyrus, medial, BA 8 |
|  | | | | |
| *Note.* This table reflects the meta-regression report assessing the effect of clinical population status on functional connectivity outcomes. All clusters were non-significant (*p* > .05). | | | | |
|  |  |  |  |  |
