# Supplementary material for: Meta-analytic evidence that mindfulness training alters resting state default mode network connectivity
Source: Sci Rep. 2022 Jul 18;12:12260. doi: 10.1038/s41598-022-15195-6 (PMC9293892; doi:10.1038/s41598-022-15195-6)
Supplement: Supplementary file 4 — Supplementary Table S4. [file 41598_2022_15195_MOESM4_ESM.docx]

**Supplemental Table S4. Literature Search Key Terms**

| (“rest*(-ing)” AND “connect*(-ivity)”)  OR (“default mode”)  OR (“default mode network”)  AND  (“mindfulness” OR “meditation” OR “MBSR” OR “MBCT”) | | | |
| --- | --- | --- | --- |
